# How noise correlation impact population code in superior colliculus: an information theoretic approach

**DOI:** 10.1186/1471-2202-14-S1-P307

**Published:** 2013-07-08

**Authors:** Saba Farbodkia, Kelly Shen, Gregg S Day, Martin Paré

**Affiliations:** 1 Center for Neuroscience Studies, Queen's University, Kingston, Ontario, K7L 3N6, Canada; 2 Rotman Research Institute, Toronto, Ontario, M6A 2E, Canada; 3 University of Toronto, Toronto, Ontario, M5S 3G4, Canada

## 

We previously showed that superior colliculus (SC) visuomovement neurons recorded while monkeys perform visual search tasks have discriminating ability that exceeds the monkey's discriminating ability, suggesting that there is substantial noise added to this neuronal population [1]. We recently began to investigate the latter issue with modeling. First, we examined the effect of noise correlation as a limiting factor on the population's overall discrimination ability. To do so, we simulated a population of singly recorded neurons while manipulating noise correlation within the range of our estimates made from simultaneously recorded pairs of neurons with overlapping receptive fields. Positive noise correlation did decrease the performance of our simulated neuronal population. Based on these results, we sought to further investigate the effect of noise correlation on the discriminating ability of simultaneously recorded pairs of neurons.

Here, we examine the effects of noise correlations on ability of simultaneously recorded pairs of neurons in SC to discriminate between two types of stimuli, the target and distractors. We use different time intervals of these recorded responses, to quantify the amount of transmitted information in two conditions: once with the cross-correlations among cells taken into consideration, and another time, considering the cell responses being independent. By comparing the amount of information between the two conditions, we can determine if the noise correlations have a role in limiting the performance of subjects compared to that of the single neurons. If the amount of information with regard to discrimination of the stimuli is larger in the joint population responses than in the sum of information in responses of individual neurons, then the noise correlations have an enhancing effect on discrimination. On the other hand, less information for the joint neuronal activity compared to the independent responses, can indicate that noise correlations are indeed one of the limiting factors that decrease the efficiency of SC in a visual search task.

We explore different time windows during the trial for this analysis, since the effects are expected to be larger, and therefore, more tractable, in time intervals that are further from saccade point, when the amounts of noise are larger.
